# Osteoglycin: An ECM Factor Regulating Fibrosis and Tumorigenesis

**DOI:** 10.3390/biom12111674

**Published:** 2022-11-11

**Authors:** Jiayida Nulali, Ming Zhan, Kaiwen Zhang, Pinghui Tu, Yu Liu, Huaidong Song

**Affiliations:** 1 The Core Laboratory in Medical Center of Clinical Research, Department of Molecular Diagnostics and Endocrinology, Shanghai Ninth People’s Hospital, Shanghai Jiao Tong University School of Medicine, Shanghai 200011, China; 2 Department of Respiration, Yangpu Hospital, Tongji University School of Medicine, Shanghai 200070, China

**Keywords:** osteoglycin, extracellular matrix, fibrosis, tumorigenesis, epithelial-mesenchymal transition

## Abstract

The extracellular matrix (ECM) is made up of noncellular components that have special properties for influencing cell behavior and tissue structure. Small leucine-rich proteoglycans (SLRPs) are nonfibrillar ECM components that serve as structural scaffolds and signaling molecules. osteoglycin (OGN), a class III SLRP, is a ubiquitous ECM component that not only helps to organize the extracellular matrix but also regulates a number of important biological processes. As a glycosylated protein in the ECM, OGN was originally considered to be involved in fiber assembly and was reported to have a connection with fibrosis. In addition to these functions, OGN is found in a variety of cancer tissues and is implicated in cellular processes linked to tumorigenesis, including cell proliferation, invasion, metastasis, and epithelial-mesenchymal transition (EMT). In this review, we summarize the structure and functions of OGN as well as its biological and clinical importance in the context of fibrotic illness and tumorigenesis. This review aims to improve our understanding of OGN and provide some new strategies for the treatment of fibrosis and cancer.

## 1. Introduction

The extracellular matrix (ECM) is a complex network composed of an array of multidomain macromolecules organized in a cell-matrix network manner and plays an important role in physiology and pathophysiology [1,2]. The ECM is made up of noncellular components with specific functions in regulating cell behavior [2]. ECM components are now widely known to mediate and modify signal transmission and are also linked to a variety of illnesses, including cardiovascular and skeletal issues, fibrosis, and cancer [3]. Of particular interest are small leucine-rich proteoglycans (SLRPs), a group of ECM components involved in matrix structural organization. These molecules have been extensively studied for their ability to bind collagen and their roles in tissue association, fibrosis, and wound repair [4,5,6,7].

SLRPs are made and secreted in the pericellular spaces, where they are eventually integrated into the ECM of the tissue [8]. Because of the diversity in their leucine-rich repeat (LRRs) cores and glycosylation patterns, SLRPs can bind several growth factors, such as the insulin like growth factor receptor (IGFR) [9], epidermal growth factor receptor (EGFR) [10], and transforming growth factor-β (TGF-β) [11]. Therefore, in addition to being an important structural component of the ECM, SLRPs have been implicated in the complex network of the cells’ ‘inside-out’ signal transduction and participate in a wide range of processes such as inflammation, atherosclerosis, and tumorigenesis, which are critical for many processes [12,13,14,15].

OGN, a class III member of the SLRP family, was isolated from bone and originally called osteoinductive factor [16]. Among a group of proteins identified as important regulatory proteins within the ECM [17], OGN is expressed in a variety of organs and has effects on bone formation [18], fibrillogenesis [19], tumorigenesis [20], and pathological processes, including vascular differentiation and remodeling [21]. This molecule is also implicated in connective tissue-related diseases [22].

Fibrosis is a disease that affects practically every tissue in the body and is defined by the buildup of ECM, which is made up of fibrous macromolecular proteins such as collagen and elastin and is becoming increasingly crucial in the evolution of fibrosis and other chronic disorders [23]. SLRPs are key components that regulate fibrosis, inflammation, and tumor growth, among other pathophysiological processes [24]. In particular, OGN expression is linked to fibrosis-related factors [25]. Early in vitro research revealed that OGN interacts with collagen via specific binding sites and delays the development of collagen fibrils [5], altering both the ECM and fibrillogenesis, with implications for tissue function, particularly in fibrotic illness [19]. Previously, a skin-fragility test revealed slightly reduced tensile strength of the skin of OGN-deficient mice compared to wild-type littermates [19,26]. This effect was attributed to a difference in collagen fibrillogenesis, where OGN-null mice displayed thicker collagen fibrils in both corneal and skin tissues [19].

Cancer is a rapidly spreading, noncommunicable disease that is a major public health threat. The study of how cellular components of the ECM originate and promote tumorigenesis has received much attention [27]. The interaction of cells with the ECM occurs through molecules regulating survival, proliferation, migration, differentiation, and polarity. As a result, throughout the progression of cancer, the ECM undergoes considerable modification [28]. ECM also causes dynamic changes in the tumor microenvironment and is involved in cancer progression [29]. Normal cells are transformed throughout this process, resulting in increased survival, aggressive proliferation, and invasion. In a rising number of investigations, SLRPs have been shown to alter cellular behavior and tumor progression through interactions with growth factors or tyrosine kinase receptors [30,31]. OGN is a glycosylated protein that participates in the development of the ECM, and is implicated in cellular processes linked with tumorigenesis, and is regulated by the tumor suppressor protein p53 [32,33]. Several studies examining the role of OGN in tumorigenesis have concluded that OGN plays an important role in forming the cancer microenvironment [20].

The current review will concentrate on the role of OGN in controlling the fibrosis and tumorigenesis processes, including a number of pathologies related to fibrosis and cancer. Because many of the effects of OGN are related to its function, the structure and functions of OGN will also be discussed.

## 2. OGN—Structure and Functions

**Figure 1:**
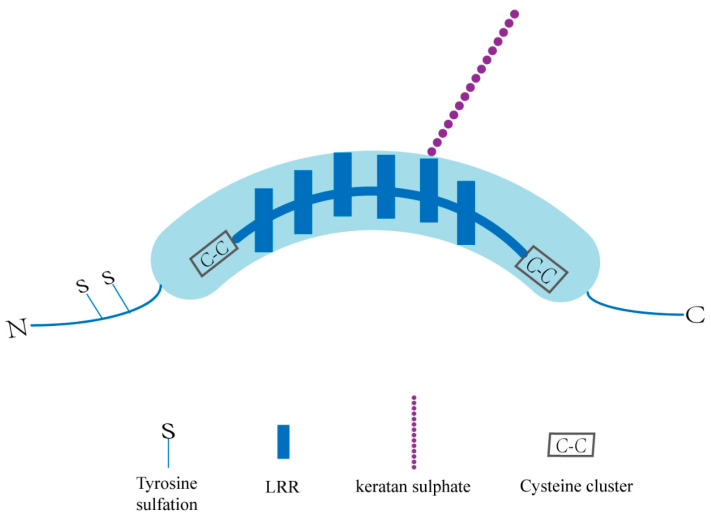
Basic structure of an OGN. KS: keratan sulphate; GAG: glycosaminoglycans; N: amino terminal; C: carboxyl terminal; LRR: leucine rich repeat motifs.

The overall structure of SLRPs consists of a core protein composed of LRRs and an N-terminal cysteine-rich cluster bound to specific glycosaminoglycans (GAG) [34,35]. OGN, a class III SLRP with numerous glycosylation sites, has a unique cysteine-rich region sequence as well as six LRRs (Figure 1). Structurally, OGN can be glycosylated to a defined level, and that adds glycosaminoglycans (GAG) and glycans to proteins in the endoplasmic reticulum (ER) and Golgi apparatus, allowing them to perform a variety of functions [36]. The GAG chains are believed to function in the maintenance of interfibrillar spacing and normal tissue hydration [37,38]. Because of differential splicing, alternative polyadenylation, and posttranslational modifications, OGN mRNA and proteins of different sizes have been identified [39,40,41]. Additionally, OGN is a single-copy gene on the human 9q22.31 chromosome that produces three mRNA transcripts, three of which are the result of differential splicing inside the first translated exon [42,43]. Because of proteolytic cleavage and glycosylation, different protein isoforms exist, whereas only one precursor protein is encoded [42]. In our previous study, the full-length OGN cDNA was identified during our establishment of the gene expression profile in the human pituitary gland (GenBank no. AF100758) [44]. A conserved consensus p53-binding DNA sequence in the first intron of the human OGN gene was validated, and p53 can activate OGN expression via this binding sequence [41]. Thus, OGN has been accepted as a direct target gene of p53 [41]. UV-responsive E-box and several interferon-stimulated response elements (ISREs), which have been identified and demonstrated to operate as positive regulators of the human OGN promoter, were also found in the promoter region of the bovine and human OGN genes [32,45].

OGN was found in a variety of tissues, including the cornea, skin, aorta, sclera, vagus nerve, and cartilage, as well as at lower levels in the ovary, cerebellum, skeletal muscle, heart, and kidney [39,46]. Additionally, post-translational modifications of the protein increase the diversity of OGN, and a higher level of functional diversity is achieved this way [42]. OGN is secreted into the ECM after post-translational modification, where it interacts with a variety of molecular targets and plays a variety of biological roles [47]. Fibrillar collagens, growth factors, and ECM molecules are examples of cell membrane receptors. As a result, ECM assembly [17] and immunity [33] can be influenced. Its functions were subsequently extended to include corneal transparency [26], fibrosis [48], and cancer biology [49].

To date, many of these breakthroughs have been the result of studies on mouse models deficient in OGN genes. Initially, the most pronounced increase in collagen fibril diameter was found in the skin of OGN-null mice [19]. Secondly, connective tissue growth factor (CTGF) reduces OGN mRNA expression, resulting in increased fibroblast proliferation. However, decreased OGN expression caused by micro-RNA(miR)-22 results in increased cardiofibroblast senescence [50]. Tumor necrosis factor-a (TNF-α) increases fibroblast OGN expression in an NF-κB/IKK-dependent manner [51], and this process is essential for the formation of fibrosis. However, downstream processes remain to be identified. High OGN expression is required for the development and maintenance of the corneal matrix as well as corneal transparency [51,52]. Bone Morphogenetic Protein 2 (BMP-2) increased osteoblast OGN expression, which also resulted in increased osteoblast differentiation and bone development [18]. Our previous study demonstrated that OGN is expressed in mouse and human pituitary tissues and regulated by pituitary transcription factor-1 (Pit-1) [53]. In addition, glucocorticoids increase OGN expression in pituitary corticotroph cells, and this upregulation could be mediated by the classic GR pathways [54], which are important for ACTH secretion and HPAA balance [55]. Extracellular OGN increases IL-1β and IL-6 expression in neurons in the hypothalamus, possibly influencing satiety [56]. Furthermore, OGN has been discovered in both local and circulating innate immune cells as OGN costained with neutrophil and macrophage markers in the myocardium [57]. As a component of the normal vascular matrix, OGN is expressed primarily in smooth muscle cells (SMCs) and perivascular fibroblasts, where it plays an important role in arteriogenesis, cellular growth [58], cell proliferation, and migration [59]. However, the physiological function of OGN has not been fully elucidated. Given the structural and functional diversity of OGN, as well as its widespread expression, it is no surprise that it is involved in a wide range of disorders.

## 3. OGN as a Regulator of Fibrosis

### 3.1. Role of OGN in Cardiac Fibrosis

**Figure 2:**
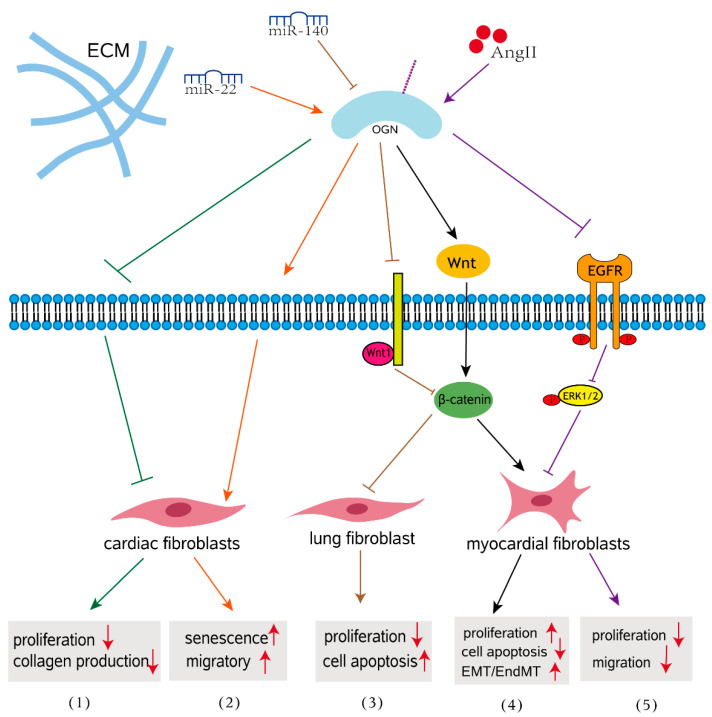
OGN signaling in fibrosis. (1) OGN reduces cardiac fibroblast proliferation and TGFβ-mediated collagen production in age-related hearts. (2) MiR-22 overexpression induced cellular senescence and promoted the migratory activity of cardiac fibroblasts by targeting OGN in the aging heart. (3) Overexpressed miR-140 inhibits pulmonary fibrosis in interstitial lung disease via the Wnt signaling pathway by downregulating OGN. (4) OGN enhances myocardial fibrosis and reverses EMT/EndMT in a mouse model of myocarditis. (5) OGN regulates cardiac myofibroblast proliferation and migration in response to AngII by suppressing EGFR signaling.

In various cardiac pathophysiologic circumstances, cardiac fibrosis is defined as an imbalance of ECM production and breakdown, which contributes to heart dysfunction [60]. Cardiac fibrosis is a process of pathological ECM remodeling, leading to abnormalities in matrix composition and quality as well as impaired heart muscle function [61]. Evidence for the involvement of OGN in the fibrosis of cardiovascular disease is abundant. A meta-analysis revealed that the blood concentration of OGN in patients with cardiovascular disease is significantly elevated compared to that in control patients, indicating that OGN may play an important role in cardiovascular disease [62]. OGN is expressed by cardiac fibroblasts, vascular smooth muscle cells, and cardiomyocytes [63,64]. The earliest studies have shown that OGN is associated with cardiac hypertrophy in a genome-wide analysis of the rat heart, and it influence left ventricular mass through modulation of the TGF-β pathway [65]. Interestingly, downregulation of OGN in aging hearts enhanced migration of cardiac fibroblasts, promoting aging associated cardiac fibrosis [65]. More specifically, compared with those with nonischemic heart failure, circulating OGN levels were significantly increased in patients with a previous history of myocardial infarction (MI) and correlated with survival, left ventricular volumes, and fibrosis [64]. In wild-type mice, adenoviral overexpression of OGN increased collagen quality, preventing heart dilatation and dysfunction after MI [64]. This finding indicates that OGN after myocardial infarction is essential for cardiac remodeling and that OGN is perhaps a potential biomarker for ischemic heart failure. Activated cardiac myofibroblasts are not only the main cell type responsible for increased interstitial collagen accumulation in fibrotic cardiac tissues [64,66], but also play a crucial role in the progression of pathological fibrotic cardiac remodeling [67]. Mice lacking OGN exhibited enhanced cardiac interstitial fibrosis and significantly more severe cardiac dysfunction following Ang II infusion than wild-type mice and presented with increased proliferative activity in the heart [68]. Disruption of OGN favors cardiac myofibroblasts (CMFs) growth and facilitates cellular motility through the LPA3/Rho/ROCK dependent MMP-2/EGFR/ERK signaling pathway, contributing to ECM deposition and cardiac dysfunction (Figure 2) [68]. These findings addressed how OGN functions in response to hypertension-associated cardiac fibrosis. In human and mouse myocarditis, epithelial-mesenchymal transition (EMT) and endothelial mesenchymal transformation (EndMT) have emerged as important sources of myofibroblasts that regulate the Wnt signaling pathway during the development of myocardial fibrosis [69,70]. In a mouse model of myocarditis, OGN gene silencing inhibited proliferation of mouse myocardial fibroblasts and suppressed EMT and EndMT by activation of the Wnt signaling pathway, thus further resulting in the alleviation of myocardial fibrosis after myocarditis [48]. This finding emphasized the role of OGN in myocardial fibrosis that develops after myocarditis. The absence of circulating OGN reduced cardiac inflammation and injury in viral myocarditis [57].

### 3.2. Impact of OGN on Fibrosis in Other Organs

In addition, overexpression of OGN contributes to the progression of interstitial lung disease (ILD) via the Wnt signaling pathway [25]. MiR-140 downregulates OGN, resulting in activation of the Wnt signaling pathway and further modulating the expression of genes associated with the progression of pulmonary fibrosis in mouse fibroblasts, including transforming growth factor beta (TGF-β1), TNF-α and CTGF included (Figure 2) [25]. TNF-α is a proinflammatory cytokine that has been extensively reported to be an important factor in the pathogenesis of pulmonary fibrosis [71]. In the normal kidney, SLRPs are secreted by renal fibroblasts and exist mainly in the peritubular space, with trace amounts present in the glomerular space [72,73]. In fibrotic renal disease, SLRPs increase and accumulate in areas of tubulointerstitial fibrosis [74,75,76]. In a diabetic mouse model, OGN was downregulated in db/db mice. Decreased OGN as well as activation of the NF-κB signaling pathway were correlated with the pathogenesis of diabetic nephropathy [77]. These results indicate that OGN may be involved in the pathogenesis of diabetic fibrotic complications and may play a beneficial role in the development of diabetic nephropathy.

Based on the evidence, OGN appears to be a crucial regulator of fibrosis. The role of OGN in the regulation of fibrosis is complex, with competing profibrotic and antifibrotic actions, including in the regulation of EMT. Thus, further research in this direction is worth considering.

## 4. OGN Function in Tumorigenesis

### 4.1. OGN in Tumor Initiation

The complex interactions between cancer cells and their microenvironment, which includes cancer-associated fibroblasts (CAFs), endothelial cells, immune cells, adipocytes, and the ECM, are known to play a role in tumorigenesis [78,79]. Numerous studies have validated the expression pattern of OGN in various types of malignancies, and the expression level of OGN has been observed to vary in different forms of cancer. In most cases, the expression of OGN is decreased in tumor tissues compared with normal tissues, as shown in squamous cervical cancer [80], gastric cancer [81], colorectal cancer [20], vaginal cancer [80], invasive ductal breast carcinoma [82], laryngeal carcinoma [83], and thyroid tumors [84]. However, in pituitary tumors and ovarian carcinoma, OGN expression is differentially dependent on the tumor type [53,85]. Furthermore, OGN is expressed in various cancer tissues and has been reported to have a positive or negative correlation with tumor progression [86,87]. In several malignancies, OGN expression is important for predicting overall survival (OS) and clinical outcomes [86,87,88]. More specifically, OGN gene expression was shown to be under the control of p53, a known tumor suppressor [41]. P53 is frequently mutated to inactivation in several tumors, including lung cancers, colorectal cancers, and breast cancers, in turn leading to the inactivation of the OGN gene [88]. Moreover, tumor-suppressor activity was observed in mouse melanoma cell lines as overexpression of OGN increased stress-triggered cell death autophagy following ER induction through LIP-mediated pathways following ER stress [89]. The stimulation of the mTOR pathway in OGN-overexpressing cells results in cell death and autophagy [78]. Similar to other SLRPs, OGN is involved in signal transduction that mediates a variety of responses in relation to cancer progression. This molecule can either promote or inhibit cancer progression, exhibiting both protumorigenic and antitumorigenic characteristics [15].

### 4.2. OGN in Celluar Progression

**Figure 3:**
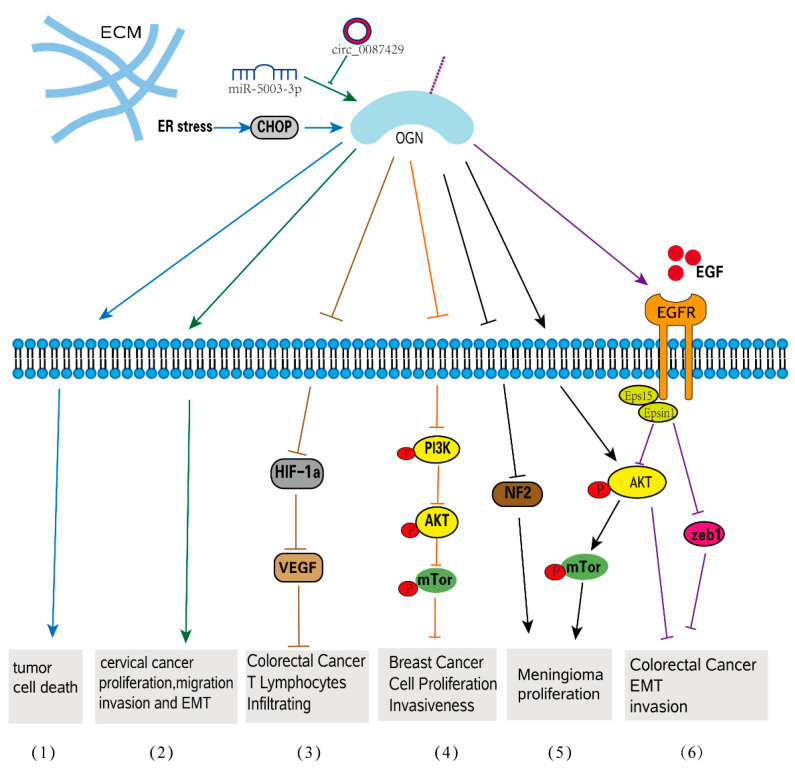
OGN signaling in tumorigenesis. (1) ER stress increases OGN expression and subsequent tumor cell death. (2) circ_0087429 can reverse EMT and inhibit the progression of cervical cancer via miR-5003-3p-dependent upregulation of OGN expression. (3) In colorectal cancer, OGN enhances T lymphocyte infiltration by inhibiting the activation of HIF-1α and significantly inhibiting the expression of VEGF. (4) OGN inhibits cell proliferation and invasiveness in breast cancer via the PI3K/Akt/mTOR signaling pathway. (5) OGN promotes meningioma development through downregulation of NF2 and activation of mTOR signaling. (6) OGN reverses EMT and invasiveness in colorectal cancer via the EGFR/Akt/zeb1 pathway.

OGN controls tumor cell proliferation by a variety of methods, either increasing or inhibiting cancer growth, according to numerous studies. A previous proteomic study found that, whereas all adenoma and cancer tissues did not express OGN, all normal mucosa did, indicating that OGN deficiency is linked to the development of colorectal cancer [88]. Furthermore, patients with high OGN expression had a significantly longer survival in colorectal cancer, and a high blood OGN level was consistently related to fewer recurrences [86]. It highlights the importance of OGN-mediated EGFR signaling in inhibiting tumor development [86]. In this cancer type, the correlation between OGN expression and T-cell densities was verified by immunohistochemistry [20]. In colorectal cancer cells, increased OGN expression hindered the activation of the transcriptional gene hypoxia-inducible factor-1 (HIF-1), which then inhibited the synthesis of vascular endothelial growth factor (VEGF), reducing T-cell tumor invasion [20]. The effect of OGN on inflammatory cell infiltration in colorectal cancer is better explained in this paper, demonstrating its function in tumor microenvironment regulation. Because of its effects on the PI3K/AKT/mTOR pathway, overexpression of OGN dramatically reduces cell growth in breast cancer cells, demonstrating that OGN is a tumor suppressor in breast cancer [90]. Meningiomas, in contrast, have significantly higher mRNA expression of OGN than other brain cancers and normal brains. Through downregulation of (neurofibromatosis type 2) NF2 and activation of mTOR/Akt signaling, OGN-overexpressing meningioma cells displayed an increased rate of cell proliferation, cell cycle activation, and colony formation when compared to control cells [87] (Figure 3). A new study found that chrysophanol, an anthraquinone with purportedly potent antitumor effects, can block OGN’s action on meningiomas in order to achieve its antitumor effect [91,92].

OGN is also important in modulating the microenvironment and tumor biology in head and neck squamous cell carcinoma, according to a protein-protein interaction analysis (HNSCC) [93]. Recently, OGN was discovered to be upregulated in dormant breast cancer cells compared to proliferative cells, serving as a marker for breast cancer and a prognostic factor for breast cancer patients [94]. OGN was also found to be significantly associated with poor survival in patients with gastric cancer (GC), and its expression increased as the cancer progressed, according to a functional enrichment analysis [95]. According to a bioinformatic analysis, OGN plays vital roles in immune surveillance and tumor progression in papillary thyroid cancer [96].

### 4.3. OGN in EMT-Related Tumorigenesis

EMT, a crucial process in which epithelial cells gradually transform into mesenchymal-like cells and lose their epithelial functionality and characteristics, is linked to tumor migration and aggressiveness [97,98]. OGN is involved in EMT-related cellular processes such as migration, invasion, and adhesion. OGN can reverse EMT by suppressing the EGFR/AKT/Zeb-1 axis, which is an indicator of increased survival and decreased cancer recurrence in colorectal cancer [86]. EGFRs are frequently activated, crosstalk with other pathophysiologies, such as EMT and carcinoma angiogenesis in human colorectal cancer, and play crucial roles in tumor development and progression [99]. By affecting EGFR dimerization, internalization, and recruitment of eps15 and epsin1 to EGFR, OGN inhibited EGFR kinase activation and attenuated the downstream activators Akt and Zeb-1, which inhibited EMT of cancer cells and decreased tumorigenesis. Overexpression of OGN significantly inhibits cell proliferation and migration/invasion and reverses EMT phenotypes in breast cancer cells through its effects on the PI3K/AKT/mTOR pathway. Many studies have shown that activation of this pathway can promote EMT conversion [100,101,102]. The epithelial-specific protein E-cadherin was upregulated in OGN-overexpressing cells compared to controls, while the interstitial proteins vimentin and N-cadherin were downregulated. OGN appears to be a tumor suppressor in breast cancer [90]. Additionally, when OGN was overexpressed in mouse hepatocarcinoma cells by extrinsic transfection, decreased migration and invasion capacity as well as decreased metastasis to peripheral lymph nodes were observed [103]. A circular RNA, named Circ 0087429, can inhibit EMT in the occurrence and development of cervical cancer by competitively binding with miR-5003-3p and regulating the expression of its target gene OGN. (Figure 3). It was discovered that, when compared to nontumor tissues, OGN expression was upregulated in serous papillary cystadenocarcinoma and endometrioid adenocarcinoma [49], but not in clear-cell ovarian carcinoma [85]. OGN expression was found to be an adverse prognostic factor for both overall and progression-free survival in ovarian carcinoma patients in a Kaplan-Meier analysis. Furthermore, high OGN expression is linked to a stronger enrichment of the EMT-related transcriptional program as well as a significant trend toward poor clinical outcomes [85].

The tumorigenic process is connected to OGN, which could be employed as a biomarker for tumor prognosis. Based on existing data and in a cancer-type-specific manner, OGN has been characterized as both an anticancer chemical and a tumor promoter, which depends on the tumor type. Therefore, OGN is proposed to be both a therapeutic target and an anticancer agent. However, the majority of studies on OGN in cancer indicate its antitumorigenic function. As a result, OGN could be a potential cancer biomarker, but its characterization in various cancers is still unknown. Table 1 summarizes the effects of OGN on various cellular functions in cancer cells.

## 5. Conclusions and Future Perspectives

The study of how components of the ECM originate and promote disease development has required substantial time and effort. The role of OGN in fibrosis and carcinogenesis, as well as the mechanism of action of OGN in these disorders, are thoroughly examined in this review. Understanding the molecular events that underpin the disease may help us better understand its characteristics and may even aid in the prevention of some chronic diseases and cancers. OGN, which belongs to the SLRPs, is a critical component of the ECM that regulates collagen fibrils and mediates cell signaling. OGN has been linked to bone development, premature labor, tumor biology, corneal transparency, and skin integrity, among other biological processes. As a major matrix proteoglycan, OGN has a critical function in the regulation of fibrosis and tumorigenesis, as discussed in this review. Increasing evidence has shown that OGN plays a role in various fibrotic diseases, especially cardiac disease. However, the role of OGN in the process of cardiac fibrosis is complicated and confusing. OGN deficiency resulted in reduced and insufficient fibrosis following ischemic heart failure [48], whereas downregulation of OGN in aging hearts facilitated the migration of cardiac fibroblasts, promoting aging-associated cardiac fibrosis [65]. Osteoglycin attenuates cardiac fibrosis by suppressing cardiac myofibroblast proliferation and migration [68] and prevents the development of age-related diastolic dysfunction during pressure overload by reducing cardiac fibrosis [104], indicating that OGN is increased as a compensatory mechanism to limit cardiac fibrosis. Therefore, we believe that OGN could be a promising therapeutic target for individuals with age-related cardiac fibrosis and ischemic heart failure. Besides. Experimental evidence has shown that activation of the Wnt signaling pathway is important in OGN-induced organ fibrosis [105]. OGN gene silencing restrained the activation of the Wnt signaling pathway, thus resulting in the alleviation of myocardial fibrosis. However, miR-140-induced inhibition of OGN exerted antifibrotic action by activating Wnt signaling in ILD. More evidence points to its antifibrotic effect, except for the role of OGN in pulmonary fibrosis. It is also worthwhile for us to think deeply about the complexity of its function. Given the importance of fibrotic diseases and the estimation that over half of deaths are associated with fibrosis [106], further investigation into the role of OGN is important.

Several recent investigations have revealed that OGN has a greater role in carcinogenesis, particularly in tumor EMT processes. OGN has also been identified as a key regulator in tumor proliferation and invasion in colorectal, breast, and ovarian cancers [85,88,90], as well as in enhancing T lymphocyte infiltration in colorectal cancer [20], indicating its role in regulating the tumor microenvironment. OGN has been identified as both an anticancer chemical and a tumor promoter, as seen in Table 1 and Figure 3. All of these results point to OGN regulation as a potential novel treatment method for preventing tumor development, metastasis, and EMT. The diverse functions of OGN in tumorigenesis may be attributed to its ability to interact with numerous components of the ECM and cellular receptors, thereby inducing intracellular signaling cascades. Nevertheless, whether OGN is involved in other tumors remains to be explored. However, it is likely that our current understanding merely scratches the surface of OGN’s several roles in fibrosis and tumorigenesis. The majority of the data are limited to OGN, with new signaling pathways being found every year. This may be an issue for further study. The review’s most important finding is that OGN has the potential to be used for effective therapeutic techniques.

Fibrotic alterations in tissue fibrosis, in a larger sense, are analogous to cancer stromal reactions and generate a tumorigenic milieu. Fibrosis may occur in conjunction with the occurrence of a tumor and may alter with the occurrence of tumorigenesis. It could be a sign that OGN is worth investigating further.

## Figures and Tables

**Table 1:** The role of OGN in cancer.

Cancer Type	Expression (Protein/mRNA)	Level of Expression	Role	Function in Cancer	Ref.
Colon cancer	Protein	Downregulated	Tumor suppressor	Suppressing invasion, migration, and EMT and prolonging OS	[86]
Colon cancer	Protein	Downregulated	Tumor suppressor	Enhancing T iymphocytes infiltrating	[20]
Breast Cancer	mRNA/Protein	Downregulated	Tumor suppressor	Inhibiting cell proliferation, invasiveness, and EMT and prolonging OS	[90]
Liver cancer	Protein	Downregulated	Tumor suppressor	Decreasing metastatic potential to peripheral lymph nodes and prolonging OS	[90]
Cervical cancer	mRNA/Protein	Downregulated	Tumor suppressor	Suppressing proliferation, migration, and invasion and reversing EMT	[49]
Ovarian cancer	mRNA/Protein	Upregulated	Oncogene	Being associated with EMT and poor prognosis	[85]
Meningioma	mRNA/Protein	Upregulated	Oncogene	Accelerating proliferation	[87]
Melanoma	mRNA/Protein	Upregulated	Oncogene	Increasing cell death	[89]
